# Observations on the Suppression of Cutaneous Eruptions in Children

**Published:** 1827-06

**Authors:** Edward Morton

**Affiliations:** Physician to the Royal Metropolitan Infirmary for Sick Children.


### *Observations on the Suppression of Cutaneous Eruptions in Children.* By Edward Morton, M.B. L.M. Physician to the Royal Metropolitan Infirmary for Sick Children.

The practice of applying astringents indiscriminately to the heads of infants, for the removal of the various eruptions to which they are subject, although very generally adopted, is by no means unattended with danger.

It is well known that applications of the above nature afford the most speedy means of repelling the eruptions in question. But this circumstance, in my humble opinion, forms an important objection to their incautious use; for I venture to affirm, from repeated experience, that discharges of any description about the heads of infants cannot be suddenly repressed, without the risk of inducing far more serious complaints.

I have had several cases of inflammation of the brain under my care during the last year, which occurred in infants upon porrigo of the scalp, which previously existed, spontaneously disappearing.

I have also seen many instances where the same disease has succeeded to cephalic inflammation, upon the appearance of which all symptoms of the latter affection have immediately ceased, and, so long as the eruption continued, did not return: but, upon its either disappearing spontaneously or being artificially removed, the primary complaint has been reproduced with increased violence. In cases of this description, the eruption is evidently beneficial, and is, in fact, a natural cure for the original disease. Does it not, indeed, exactly resemble the remedial means we ourselves adopt in such cases, when we produce a discharging surface by means of blisters and irritating dressings?

I have also met with other cases in which inflammation of the brain has supervened (where no previous symptoms of it had existed,) upon the intentional repulsion of eruptions on the head, by means of astringent ointments, or other similar applications. It is not long since a child, who had been attended by a surgeon for porrigo of the scalp, and which had just been removed by an ointment of the foregoing description, was transferred to my care for a fresh complaint; the medical attendant himself observing at the time, that it was evidently labouring under "inflammation of the investing membranes of the brain," with which opinion I perfectly coincided. Upon minutely inquiring into the history of the case, I found that the child never showed any symptoms of the affection of the head until after the eruption had begun to be repressed by the local treatment employed. The following case occurred to myself very lately.

An infant, thirteen months old, was brought to me at the Dispensary, affected with porrigo of the face and head: its general appearance did not indicate any tendency to cephalic disease. I merely ordered some opening powders of Rhubarb and Calomel. After a few days, the child was again brought. I now prescribed an ointment composed of equal parts of Unguent. Hydrarg. Nitrat. and Ceratum Cetacei, to be applied in the usual manner; the powders being occasionally repeated. When the mother next came, she informed me that the child had been constantly drowsy, and had laid its head down ever since I last saw her; but that the eruption was " much better." I now found that the head was beginning to be affected in consequence of the recession of the eruption; and therefore advised the use of the ointment to be immediately suspended, the parts affected to be merely washed twice a-day with warm milk and water, and the aperient powders to be repeated. Upon the next visit, I was told that, as soon as the ointment was discontinued, the child immediately regained its usual liveliness, and has been going on well ever since.

But it is unnecessary to adduce further evidence on this head; every practitioner, who has had much experience in the treatment of infantile complaints, must have repeatedly observed many similar cases. I shall, therefore, proceed at once to the chief object of the present paper, which is to suggest the necessity of attending to the practical cautions to be deduced from the foregoing facts.

From these, and from the consideration of the nature of the circulation in the head, the following conclusions, I think, may be fairly and usefully drawn:—

1. That, in all cases of cutaneous eruptions upon the heads of infants, (particularly if extensive,) danger may arise from their artificial repulsion.

2. That, in cases where eruptions have occurred upon the scalp of infants subsequently to cephalic disease, dangerous or fatal consequences will most probably ensue, upon their intentional removal by local treatment.

3. That as astringent ointments, and other applications of a similar nature, are found by experience to have the power of speedily repelling the eruptions in question, they should not be employed, without their effects being carefully watched, and their evil tendencies promptly guarded against.

The treatment of these diseases, therefore, in infants should in every case be commenced with purgatives, and repellent applications should not be made use of without due caution; such as may be selected being at first extremely mild, and afterwards gradually increased in strength. If the patient, during their employment, should become drowsy, and sleep much, or lay its head constantly down, (a sure indication of the commencement of affections of the head in infants,) they should be immediately discontinued, and purgatives be freely employed.

In cases where porrigo has attacked the scalp subsequently to cerebral inflammation, it will seldom be prudent to employ local applications at all, the cure being more safely accomplished by purgatives and alteratives.

15, *Eaton-street, Grosvenor-place;*
*April* 1827.

